# Psychometric Properties and Measurement Invariance of the Brief Symptom Inventory-18 Among Chinese Insurance Employees

**DOI:** 10.3389/fpsyg.2018.00519

**Published:** 2018-04-18

**Authors:** Mingshu Li, Meng-Cheng Wang, Yiyun Shou, Chuxian Zhong, Fen Ren, Xintong Zhang, Wendeng Yang

**Affiliations:** ^1^Department of Psychology, Guangzhou University, Guangzhou, China; ^2^The Center for Psychometrics and Latent Variable Modeling, Guangzhou University, Guangzhou, China; ^3^The Key Laboratory for Juveniles Mental Health and Educational Neuroscience in Guangdong Province, Guangzhou University, Guangzhou, China; ^4^Research School of Psychology, The Australian National University, Canberra, ACT, Australia; ^5^School of Education and Psychology, University of Jinan, Jinan, China

**Keywords:** psychometric properties, Brief Symptom Inventory-18, bi-factor model, measurement invariance, Chinese insurance professionals

## Abstract

This study aimed to examine the psychometric properties and factorial invariance of the Brief Symptom Inventory-18 (BSI-18). Confirmatory factor analyses (CFAs) were performed to verify the BSI-18’s factor structure in a large sample of Chinese insurance professionals (*N* = 2363, 62.7% women; age range = 19–70). Multigroup CFA were performed to test the measurement invariance of the model with the best fit across genders. In addition, structural equation modeling was conducted to test the correlations between the BSI-18 and two covariates – social support perception and grit trait. Results indicated that the bi-factor model best fit the data and was also equivalent across genders. The BSI-18’s general factor, and somatization and depression dimensions were significantly related to social support perception and grit trait, whereas the anxiety dimension was not. Overall, our findings suggested that the BSI-18’s can be a promising tool in assessing general psychological distress in Chinese employees.

## Introduction

The Brief Symptom Inventory-18 (BSI-18; Derogatis, 2001) is an 18-item self-report checklist, a common screening tool for psychological symptoms adapted from the Symptom Checklist-90-Revised (SCL-90-R; Derogatis, 1977) and BSI-53 (Zabora et al., 2001). Previous studies have found that the BSI-18 was highly correlated with its parent instruments—the SCL-90-R and BSI-53. Although the SCL-90-R and BSI-53 have been used extensively in clinical and community samples, both have complicated structural dimensions and large numbers of items. The BSI-18 with 18 items only was developed to more effectively obtain the most critical information about psychiatric symptoms.

The BSI-18’s brief items improved test efficiency to some extent; however, the previous findings regarding the factor structure of the BSI-18 were inconsistent. Using a Latina-speaking sample, Prelow et al. (2005) found that a single-factor model resulted from the exploratory factor analysis (EFA) was the best and most concise model. However, the authors also found that a hypothetical three-factor model fit the data reasonably well when performing confirmatory factor analyses (CFAs) using cross-validation subsamples (Prelow et al., 2005). In the three-factor model, the BSI-18 items were equally distributed to represent the three-factors of depression, anxiety, and somatization (Wiesner et al., 2010). On the other hand, Andreu et al. (2008) found a four-factor structure in a non-clinical sample of 1134 subjects (Andreu et al., 2008). Two of these four-factors (I and II) contained the same items of somatization and depression dimensions. The other two-factors had items of from the initial anxiety factor. One included a group of items assessing distress and widespread nervousness, and another included three items assessing panic symptoms.

In recent years, the bi-factor model has been increasingly popular in mapping the constructs of psychopathological scales, for instance, the Psychopathy Checklist-Revised (Flores-Mendoza et al., 2008) and the Beck Depression Inventory (Al-Turkait and Ohaeri, 2010). The bi-factor measurement structure can be an effective method for modeling multidimensional measurement tools (Reise, 2012). In the process of measuring psychological symptoms, the bi-factor model not only measures the overall situation but also places a secondary load on special symptoms represented by specific dimensions. It has been an increasingly popular view that a bi-factor structure exists between psychiatric symptoms and disorders, where both common and specific components play an important role (Watson, 2005; Thomas, 2012). It has been found that SCL-90-R and BSI-53 had bi-factor model structures (Vassend and Skrondal, 1999; Urbán et al., 2014). The bi-factor model, however, has not been tested for the BSI-18.

Another question surrounding the BSI-18 is whether the instrument has universal applicability among various non-clinical samples, ethnicities, and genders. Preceding studies demonstrated that the BSI-18 is a widely adopted measure with high internal consistency and test–retest reliability in clinical research areas (Wang et al., 2013). However, only one study tested and supported a three-factor model (somatization, depression, and anxiety) of the BSI-18 in Chinese-speaking population using a clinical sample of substance users (Wang et al., 2013). It is unclear whether the BSI-18 self-report version is similarly applicable to non-clinical samples in China.

Previous cross-cultural studies have focused on samples between different ethnicities. To determine the factorial structure and measurement invariance across races/ethnicities, Prelow et al. (2005) emphasized the need for strict invariance testing of the BSI-18 through multigroup CFA. Wiesner et al. (2010) applied a multigroup CFA to evaluate factorial invariance of the BSI-18 in women across multiple ethnicities and the three-factor model only achieved partial metric invariance. From a cross-cultural standpoint, psychological symptoms are sometimes manifested and expressed differently across populations (Wiesner et al., 2010). Each cultural group manifests its specific expression under influences of a typical language format, traditional culture, and educational background. Evaluating the measurement invariance of the BSI-18’s self-report version in a Chinese non-clinical sample can be helpful for cross-cultural research.

Many epidemiological investigations have demonstrated that women’s incidence of emotional disorders, anxiety disorders, and affective psychosis is higher than men’s (Urbán et al., 2014). In a study of insurance employee samples, Dai (2003) found that male insurers were more serious than women in terms of obsessive-compulsive and psychoticism mental health problems. Differences between men and women make it particularly important to verify the measurement model’s gender invariance. To date, the BSI-18’s measurement invariance across gender is scarcely known, especially among non-Western people.

### Mental Health in Chinese Insurance Employees

Insurance industry in mainland China faced with the pressure of external competition and self-development due to the disadvantages of being late starters (Yang, 2013). Huge organization, numerous labor, complex personnel, and high performance requirement along with high working pressure lead to high employee turnover rate and various psychological problems (Yang, 2013). Existing evidence has showed that these psychological problems correlated with social factors and personal traits, especially in insurance staff samples (Dai, 2003). It has been found that psychological distress was influenced by both perceived social support and personality traits among various professional groups (Williams et al., 2002). A lack of social support and sense of belonging have been associated with a person’s vulnerability to depression (Williams et al., 2002).

For example, grit is one important trait that may be associated with the success in insurance employees (Ling et al., 2001). Grit trait refers to firm and persistent for a long time with unswerving determination (Duckworth et al., 2007). Whether an insurance employee can tolerate high frustration and work efficiently under pressure all the time will determine his or her success as well as psychological conditions (Ling et al., 2001).

With regards to social factors, previous study shows that the level of social support of insurance employee significantly influenced their psychological well-being (Dai, 2003). For instance, severity of depressive symptoms and frequency of suicidal ideation showed negative significant correlations with low levels of social support (Zhang et al., 2010). These studies predicted, to a certain extent, correlation between specific dimensions (depression and suicide) of psychiatric symptoms and external criteria.

### Objective of the Study

This study aimed to examine the BSI-18’s psychometric properties in a large sample of Chinese insurance employees. The first goal was to examine the BSI-18’s factor structure. CFA were used to examine five hypothetical models: the original single-factor model, three-factor model, four-factor model, and bi-factor model (i.e., the three and four-factor model with one general factor). The second goal was to test measurement invariance of the BSI-18’s best-fitting model across genders using the multigroup CFA. Finally, we want to explore the manner in which the general factor (can be considered as an overall mental health status) and dimensions (specific psychiatric symptoms) were related to the social and personality covariates. Specifically, criterion validity of the BSI-18 bi-factor model was examined using structural equation modeling (SEM).

## Materials and Methods

### Participants

Participants were 2,363 insurance employees from 39 insurance companies in Guangdong Province, China. Their mean age was 35.14 (*SD* = 8.985; age range = 19–70), and 62.7% of the participants were women. Approximately 60.3% of the participants were married, and 65.4% of the participants had attained higher education (see **Table 1** for more information).

**Table 1:** Sample demographic statistics.

	Category	Frequency	Percentage
Gender	Male	865	36.6
	Female	1481	62.7
	Unreported	17	0.7
Nation	Han nationality	2275	96.3
	Other	62	2.6
	Unreported	26	1.1
Age	19–30	820	34.7
	31–40	836	35.4
	41–60	624	26.4
	>60	5	0.2
	Unreported	78	3.3
Education background	≤High school	800	33.9
	Junior college	962	40.7
	Master degree and above	583	24.7
	Unreported	18	0.8
Marital status	Unmarried	760	32.2
	Married	1425	60.3
	Other	140	5.9
	Unreported	38	1.6
Total		2363	100


### Measures

#### Brief Symptom Inventory-18

The BSI-18 (Derogatis, 2001), a brief self-report version of the 53-item BSI (Derogatis, 1993), was developed to assess general psychological distress in clinical and community populations. The BSI-18 requires participants to evaluate the extent of distress or annoyance they had experienced. Responses were rated on a five-point Likert-type scale, ranging from 1 (not at all) to 5 (very much). The questionnaire’s global score summed up all the 18 items. Internal consistency reliability for the present sample was good (Cronbach’s alpha = 0.947, 0.867, 0.859, and 0.907 for BSI total, Somatization, Depression, and Anxiety respectively).

#### Grit-8

The Grit-8 (Duckworth and Quinn, 2009), was an eight-item self-report measure that comprises eight items over two-factors, i.e., consistency of interests and perseverance of effort. These eight items were rated on a five-point Likert scale, ranging from 1 (not at all like me) to 5 (very much like me). Items 1, 3, 5, and 6 scored negatively; items 2, 4, 7, and 8 scored positively. In the current sample, the Cronbach’s alpha was 0.738 for the total scores, indicating good reliability.

#### Perceived Social Support Scale

The Perceived Social Support Scale (PSSS; Zimet et al., 1990) was a self-report instrument that measures how an individual comprehends various sources of social support, such as family and friends; the total score reflected the total degree of social support that individuals received. The PSSS comprises eight items rated on a seven-point Likert scale, ranging from 1 (not at all true) to 7 (definitely true). For this study, we selected eight items from the family and friend support dimensions. In this study, internal consistency was good for the PSSS total (α = 0.900) and two subscales (α = 0.875 for family support and α = 0.870 for friend support).

### Procedures

Participants completed aforementioned self-report questionnaires during their company’s morning meeting (administration time was approximately 30 min). The survey was administered by a trained research assistant (RA). The RA provided a general instruction of the survey before the participants started the survey. Participants could ask the RA for clarification if they did not understand any parts of the questionnaire. This study was carried out in accordance with the recommendations of Human Subjects Review Committee at Guangzhou University. All subjects gave written informed consent in accordance with the Declaration of Helsinki.

### Data Analysis Strategy

The CFA was performed separately to test five-factor structures, including the single-factor model, three-factor model, four-factor model, and two bi-factor models. Items were treated as categorical variables; thus, robust weighted least squares with mean and variance adjustment (WLSMV) was used in model estimation (Flora and Curran, 2004). Additionally, robust maximum likelihood estimator was employed to obtain the Bayesian information criterion (BIC) value for comparing the non-nested models. Model fits were evaluated using chi-squares, root-mean-square error of approximation (RMSEA), the Tucker-Lewis Index (TLI), the comparative fit index (CFI), and the BIC. Conventional guidelines indicate that an RMSEA value ≤ 0.08 indicates acceptable model fit and a value ≤ 0.05 indicates good model fit. Moreover, CFI and TLI ≥ 0.90 indicate adequate model fit (Kline, 2010). In addition, the ΔBIC value of the two models was greater than 10, indicating that the model with a smaller BIC showed a better model fit (Kuha, 2004).

To further evaluate the bi-factor models, coefficient omega hierarchical (ω_H_), the hierarchical omega subscales (ω_HS_) and the explained common variances (ECVs) were calculated to examine whether the specific factors provide utility beyond the general factor (based on the factor loadings) using the “psych” package (version 1.7.8; Revelle, 2017) in R statistical software (R Core Team, 2017). The proportion of variance in total scores estimated by ω_H_ can be attributed to a single general factor (e.g., Zinbarg et al., 2006), while the reliability of a subscale (or factor) score was reflected by ω_HS_ after controlling for the variance due to the general factor (Reise et al., 2013). When the coefficient ω_H_ is higher than 0.80, total scores can be regarded as unidimensional because of the most reliable is due to a single common factor (Rodriguez et al., 2016). Meanwhile, the large coefficient ω_H_ (>0.80) indicates that the vast majority of reliable variance imputing to a specific factor rather than a general factor (Reise et al., 2013).

To test the measurement invariance, the best-fit model resulted from the CFA was initially assessed in both male and female groups separately. Configural invariance can be indicated by that the model fits both genders equally well. Next, metric invariance and scalar invariance were tested by constraining factor loadings and thresholds of the factor models. A DIFFTEST was used to compare improvement in fit between nested models. Notably, the chi-square test was easily affected by sample size so that with increased sample size, even small differences resulted in significant differences. Thus, this research adopted the CFI (ΔCFI) difference numerical model fit indexed to evaluate measurement invariance (Cheung and Rensvold, 2002). According to Cheung and Rensvold (2002), the equivalent model is considered to be acceptable when ΔCFI ≤ 0.010 and ΔTLI ≤ 0.010.

Finally, the correlations among the factors of the BIS-18 and external criteria variables were examined using a SEM. This study used latent variables to compare observed variables and examined relations among constructs without measurement error (Oh et al., 2004). All models were performed by Mplus 7.4 (Muthén and Muthén, 1998–2010).

## Results

### Descriptive Statistics

Descriptive statistics and skewness and kurtosis for all key variables were included in **Table 2**. Due to the large values of the skewness and kurtosis, it was necessary to treat the BSI-18 variables as categorical instead on interval. Thus, we used the WLSMV to estimate models.

**Table 2:** Descriptive statistics and skewness and kurtosis for all scales included.

	M(*SD*)	Skewness	Kurtosis
BSI1	1.44 (0.745)	2.084	5.109
BSI2	1.65 (0.912)	1.477	1.875
BSI3	1.72 (0.986)	1.407	1.429
BSI4	1.27 (0.684)	3.064	10.042
BSI5	1.68 (1.038)	1.683	2.217
BSI6	1.88 (1.045)	1.261	1.087
BSI7	1.41 (0.816)	2.322	5.443
BSI8	1.62 (0.981)	1.789	2.745
BSI9	1.49 (0.924)	2.127	4.142
BSI10	1.23 (0.661)	3.487	13.252
BSI11	1.52 (0.914)	2.012	3.806
BSI12	1.37 (0.817)	2.655	7.149
BSI13	1.26 (0.679)	3.219	11.338
BSI14	1.54 (0.943)	1.989	3.610
BSI15	1.47 (0.862)	2.209	4.941
BSI16	1.51 (0.892)	2.041	4.033
BSI17	1.13 (0.575)	5.096	27.325
BSI18	1.34 (0.787)	2.824	8.332
BSI-18 total	26.15 (10.903)	2.290	6.307
GRIT total	29.88 (5.222)	-0.122	-0.280
PSSS total	39.78 (8.629)	-0.443	0.502


*BSI-18 total, Brief Symptom Inventory-18 total; GRITT, grit total; PSSST, perceived social support total.*

### Factor Structure

**Table 3** exhibits fit indices of five competing models for the polychromic correlation matrix of the BSI-18 in the whole sample. As depicted in **Table 3**, all five hypothetical models exhibited good fit to the data (CFIs > 0.90, TLIs > 0.90). Overall, the bi-factor model provided the best fit to this data among these five alternative models (WLSMVχ^2^ = 957.934^∗^, *df* = 117, CFI = 0.985, TLI = 0.980, RMSEA = 0.055, BIC = 55923.251). In the model, the general factor and three dimensions containing somatization, depression, and anxiety factors were all considered. Because the fit statistics such as CFI were similarly good for the five models (CFI values all greater than or equal to 0.950), the BIC value was used for further verification. The ΔBIC value between the three-factor bi-factor model and four-factor model was189.733, indicating that the smaller value, i.e., the three-factor bi-factor model, shows better fit (see **Table 3**). Due to the items 3 and 6 were not fully representative of anxiety dimension and item 2 of the depression dimension, we tried to re-specify a new model in which items 2, 3, and 6 don’t loading on specific factor. Difference testing result indicated a worse model fit [Δχ^2^ = 47.255, Δ*df* = 3 (*p* < 0.001)]. Thus, we still used the original three-factor bi-factor model as the best model.

**Table 3:** Goodness-of-fit indices for the five tested models of the BSI-18.

Model	WLSMVχ^2^	*df*	Δχ^2^	Δ*df*	TLI	CFI	RMSEA (90% CI)	AIC	BIC	ΔBIC
Single-factor	2678.274^∗^	135	–	–	0.949	0.955	0.089 (0.086, 0.092)	56378.054	56896.955	–
Three-factor	1631.443^∗^	132	1046.831	3	0.969	0.973	0.069 (0.066, 0.072)	55674.591	56210.789	686.166
Four-factor	1538.652^∗^	129	92.791	3	0.970	0.975	0.068 (0.065, 0.071)	55559.49	56112.984	97.805
Three-factor bi-factor	**957.934^∗^**	**117**	**580.718**	**12**	**0.980**	**0.985**	**0.055 (0.052, 0.058)**	**55181.919**	**55923.251**	**189.733**
Four factor bi-factor	968.816^∗^	117	10.882	0	0.980	0.985	0.056 (0.052, 0.059)	55195.282	55967.684	44.433


*WLSMV, weighted least squares with mean and variance adjustment; df, degrees of freedom; TLI, Tucker-Lewis Index; CFI, comparative fit index; Δχ^*2*^, change in χ^*2*^ relative to the preceding model; Δdf, change in degrees of freedom relative to the preceding model; ΔCFI, change in comparative fit index relative to the preceding model; ΔTLI, change in Tucker-Lewis Index relative to the preceding model; RMSEA, root-mean-square error of approximation; AIC, Akaike Information Criterion; ΔAIC, change in Akaike information criterion relative to the preceding model; BIC, Bayesian information criterion; ΔBIC, change in Bayesian information criterion relative to the preceding model. ^∗^*p* < 0.05. The best fitting model was in bold.*

The ω_H_ for the general factor was 0.87, and the ω_HS_ for somatization factor was 0.28, for anxiety factor was 0.17, and for depression factor was 0.04. In addition, the ECV was 80%. The bi-factor model’s standardized factor loadings were presented in **Table 4**.

**Table 4:** The standardized factor loadings for the BSI-18 bi-factor model.

		18 items			
		
		Somatization	Depression	Anxiety	General
Item 1	Faintness	0.390***			0.633***
Item 4	Pains in chest	0.537***			0.701***
Item 7	Nausea	0.449***			0.672***
Item 10	Trouble getting breath	0.499***			0.767***
Item 13	Numbness	0.427***			0.743***
Item 16	Feeling weak	0.193***			0.797***
Item 2	Feeling no interest in things		0.056*		0.729***
Item 5	Feeling lonely		0.077**		0.801***
Item 8	Feeling blue		0.048*		0.875***
Item 11	Feeling of worthlessness		0.473***		0.751***
Item 14	Feeling hopeless about future		0.468***		0.763***
Item 17	Suicidal thoughts		0.161***		0.812***
Item 3	Nervousness			-0.251***	0.853***
Item 6	Feeling tense			-0.064*	0.825***
Item 15	Feeling restless			0.056**	0.883***
Item 9	Suddenly scared			0.202***	0.888***
Item 12	Spells of panic			0.311***	0.895***
Item 18	Feeling fearful			0.210***	0.872***


*^∗^*p* < 0.05; ^∗∗^*p* < 0.01; ^∗∗∗^*p* < 0.001.*

### Measurement Invariance

To ensure that the three-factor bi-factor model provided adequate fit in each group, we first examined it separately for males and females. Results indicated that the bi-factor model fit the two groups well (see **Table 5**). Then the metric invariance model, in which item factor loadings were constrained to be equal, was tested. Results indicated negligible gender differences in model fits (ΔCFI ≤ 0.01). Finally, the scalar invariance was tested by further constraining the thresholds to be equal across the two gender groups. The scalar invariance was achieved with a ΔCFI = +0.003.

**Table 5:** Fit indices for measurement invariance.

Model	WLSMVχ^2^	*df*	Δχ^2^	Δ*df*	TLI	CFI	ΔTLI	ΔCFI	RMSEA (90% CI)
Bi-factor boys	447.379^∗^	117	–	–	0.982	0.986	–	–	0.057 (0.052, 0.063)
Bi-factor girls	573.146^∗^	117	–	–	0.982	0.986	–	–	0.051 (0.047, 0.056)
A-Metric invariance	956.841^∗^	266	–	–	0.986	0.988	–	–	0.047 (0.044, 0.050)
B-Scalar invariance	846.772^∗^	320	108.271	+54	0.991	0.991	+0.005	+.003	0.038 (0.034, 0.041)


*WLSMV, weighted least squares with mean and variance adjustment; df, degrees of freedom; TLI, Tucker-Lewis Index; CFI, comparative fit index; RMSEA, root-mean-square error of approximation; Δχ^*2*^, change in χ^*2*^ relative to the preceding model; Δdf, change in degrees of freedom relative to the preceding model; ΔCFI, change in comparative fit index relative to the preceding model; ΔTLI, change in Tucker-Lewis Index relative to the preceding model. ^∗^*p* < 0.05. Chi-square difference test with WLSMV estimation is different from the conventional chi-square difference test.*

### Criterion Validity

The SEM exhibited mediocre fit to the data (CFI = 0.847, TLI = 0.807). The general BSI factor negatively correlated with all factors of the Grit, the correlation coefficients ranged from -0.239 to -0.374 (*p* < 0.001; see **Table 6** for details). On the other hand, the somatization factor was positively and significantly related to all factors of GRIT. The depression subscale had negative correlations with Grit total and Grit Effort factor (see **Table 6**). No significant correlations were found between the anxiety factor and the Grit.

**Table 6:** Correlations between Grit, PSSS, and the BSI-18 general and dimension factors.

	GRIT			PSSS		
		
Factor	GRIT total	Effort	Interest	PSSST	Family	Friend
General BSI	-0.374***	-0.239***	-0.356***	-0.331***	-0.293***	-0.298***
Somatization	0.082***	0.068**	0.057**	0.096***	0.075**	0.089***
Depression	-0.039*	-0.020	-0.044**	-0.031	-0.024	-0.037*
Anxiety	0.011	0.006	0.011	0.003	0.004	0.004


*^∗^*p* < 0.05; ^∗∗^*p* < 0.01; ^∗∗∗^*p* < 0.001; GRITT, grit total; PSSST, perceived social support total.*

For PSSS, both the general BSI factor and the depression factor showed the strongest negative relation with all PSSS factors, the correlations coefficients ranged from -0.293 to -0.331 (*p* < 0.001). On the other hand, somatization was positively related to all PSSS factors (see **Table 6**). Anxiety factor was not significantly related to PSSS.

## Discussion

This study aimed to test the BSI-18’s factor structure and measurement invariance in a large sample of Chinese employees. The BSI-18’s bi-factor model best fit the present data. The MI tests indicated that the BSI-18 was equivalent for males and females. The results also reveal significant correlations between BSI-18 scores and grit trait and social support.

Although the one-, three-, and four-factor models achieved satisfactory fit, the bi-factor models outperformed the other three models. Moreover, the three-factor bi-factor model better fit the data than four-factor bi-factor model considering the model conciseness, and three-factor bi-factor model was chosen in the follow research. The bi-factor model consists of a general factor (General BSI) that accounted for covariation among all indicators of the comprehensive mental health level and three specific factors (somatizaton, depression, and anxiety) accounting for variance beyond the general factor in covariation among specific factor indicators (Ward et al., 2015). The current results for the bi-factor model supported the bi-factor structure of psychiatric symptoms, providing general and specific areas of composition. The bi-factor model considers the general mental health status (General BSI) while accounting for the three specific symptoms. Future studies may consider cross-cultural MI tests to clarify the cultural differences in the factor models. From another point of view, the three domain-specific components of the bi-factor model and discriminant validity demonstrated the panic factor was a product of over extraction (Recklitis et al., 2006); this is in accordance with the result of Derogatis (2001) and concludes that panic may be associated with broader anxiety symptoms.

The current finding also provided evidence for the measurement invariance of the BIS-18 across the male and female samples. The three levels of measurement invariance – configural, metric, and scalar invariance were all achieved in the present study, indicating that the BIS-18 may measure the constructs equally across the two genders.

Finally, we tested the potential covariates that may contribute to the mental conditions measured by the BSI-18. Moderate but significantly negative relations of the general BSI with grit and perceived social support were observed. This finding is consistent with the literature that the grit trait and social support were important for individuals’ mental health conditions (Williams et al., 2002; Dai, 2003). For the three dimensions beyond the general factor, the depression dimension showed modest negative correlation with grit trait and perceived social support. This is in line with the previous finding that suggested low levels of social support may result in higher level of depressive symptoms (Williams et al., 2002) and frequency of suicidal ideation (Zhang et al., 2010).

In contrast, the somatization dimension had positive correlations with the two covariates. The positive correlations of somatization with grit trait and social support may be explained by the cultural influences. Traditional Chinese culture seems to discourage people from expressing their feelings directly; thus, somatization is an alternative way to express emotional disorders (Kleinman, 1982; Cheung, 1995). When there is sufficient social support and grit characteristics, people may fear expressing their psychological distress overtly. This leads to the positive correlations of somatization with social support and grit trait. Another likely reason is that the use of the bi-factor model requires the consideration of the common differences between dimensions (Reise, 2012), and these differences can lead to cross suppression effect (Patrick et al., 2007). The correlations between anxiety dimension and grit trait and social support in this sample were not significant. In other words, all factors are included in the structural equation model, and the direction of the relationship can be reversed when these factors are tested separately.

### Clinical Significance

Cultural factors, including ethnic identity and cultural values, influence an individual’s idiomatic expression of psychological distress, conceptualization of psychological problems’ etiology, and subsequent help-seeking behavior (Torres et al., 2013). The findings in the present study have suggested that Chinese may express psychological problems via somatic symptoms. This is important in for clinical research that aims to measure mental health conditions, and for clinical practice in which how clinicians better assess patients’ problems. Clinicians may encourage Chinese patients to isolate the influence of cultural beliefs to be aware of and identify emotional problems, which may also facilitate the patients’ help-seeking behaviors. Second, as we observed, both social support and grit are associated with general mental health among the insurance employees. This implied that specific treatment options may be developed and used for the insurance practitioners. For instance, group career guidance will be affected during the morning session to alleviate employees’ occupational stress. Finally, focusing on various special symptoms, the concurrency of various adverse symptoms and the individual’s overall psychological state should be simultaneously considered during clinical research and intervention.

### Limitations

Some limitations need to be acknowledged. Because expression of psychopathology may be restricted by specific cultural backgrounds, the research’s lack of foreign samples as reference groups may be problematic. From this perspective, assessing psychological symptoms of a specific cultural norm to strengthen cross-cultural research is essential. In terms of relationships with external criteria, more other social and behavioral manifestations can be investigated, such as family-to-work conflicts, sources of social pressure, social desirability, days out of work, and so forth. Moreover, the general factor of bi-factor model maybe represent a statistical artifact factor, only in theory, but the factor loadings on general factor were large enough in our data, so it is couldn’t be caused by artifact effect or method effects.

In sum, this study suggested that the BSI-18 is a reliable and valid general psychological distress measurement instrument that can be extended to Chinese insurance employees. The bi-factor model better represented the BSI-18’s underlying structure. Meanwhile, Chinese men and women shared a common understanding of psychological distress as measured by the BSI-18. Furthermore, this study highlighted the importance of assessing the general factor and viewed the mental health of insurance practitioners as a holistic approach rather than focusing on individual dimensions while excluding the artifact effect or method effects.

## Author Contributions

ML and CZ made substantial contribution to the analysis and to the interpretation of the data, drafted the manuscript, provided final approval for the version to be published, and agreed to be accountable for all aspects of the work in ensuring that questions related to the accuracy or integrity of any part of the work are appropriately investigated and resolved. M-CW made substantial contributions to the conception and the design of the study, drafted the manuscript, provided final approval for the version to be published, and agreed to be accountable for all aspects of the work in ensuring that questions related to the accuracy or integrity of any part of the work are appropriately investigated and resolved. FR, XZ, YS, and WY helped out in the interpretation of data for the work, revised the manuscript critically for important intellectual content, provided final approval for the version to be published, and agreed to be accountable for all aspects of the work in ensuring that questions related to the accuracy or integrity of any part of the work are appropriately investigated and resolved.

## Conflict of Interest Statement

The authors declare that the research was conducted in the absence of any commercial or financial relationships that could be construed as a potential conflict of interest.
